# The Nigerian youth knowledge, Attitude and Practice (KAP) towards the national drive against the Spread of COVID-19: An online cross-sectional Survey

**DOI:** 10.12688/f1000research.121826.2

**Published:** 2024-01-09

**Authors:** Boluwaji Jaiyesimi, Toba Bamitale, Babajide Diyaolu, Kolawole Alabi

**Affiliations:** 1 Sports Science Unit, Afe Babalola University, Ado Ekiti, Ekiti, 23405, Nigeria; 2 Family Medicine Unit, Afe Babalola University, Ado EKiti, Ekiti State, 23405, Nigeria

**Keywords:** Perception, health practices, adolescents, knowledge, attitude, COVID-19

## Abstract

**Background:**

The global impact of coronavirus disease 2019 (COVID-19)has led to the need to prioritise public health campaign by all stakeholders among diverse population groups. This study investigated the dispositions of Nigerian adolescents towards the prevention of the spread of COVID-19.

**Methods:**

The young adolescents (n=1,529) included in this study, were between the ages of 15 and 26 years from Afe Babalola University. Students were contacted through their various colleges and residential hostels to complete the questionnaire *via* a shared link. All students included, voluntarily participated in this cross-sectional study by completing the adapted COVID-19 knowledge, attitude and practice (KAP) questionnaire. The collected data were analysed to show the level of knowledge, attitude and preventative practices and also to test for significant association between gender and variables for the study. The study was conducted between March 23-April 25, 2021.

**Results:**

The outcome of the findings showed that students with families with 5-10 members showed significant association with knowledge accuracy on COVID-19 (χ ^2^ = 6.077, p = 0.044). There was a significant association between gender and the need to report the suspected case of COVID-19 infection to the health authorities (χ ^2^ = 14.075, p = 0.001) with more females likely to report a suspected case. More females (59.2%) were significantly (χ ^2^ = 8.904, p = 0.012) involved in the practice of social distancing or home quarantine as a preventive measure.

**Conclusions:**

This study showed evidence of high prevalence of knowledge related to COVID-19 in majority of the study participants. This is a pointer to the efficacy and success of present public health campaigns in Nigeria.

## Introduction

Coronavirus disease 2019 (COVID-19) has evolved into a major global public health concern ( Habib *et al.,* 2021). Since the 31st of December 2019, there has been over 209 million cases of COVID-19 reported worldwide, including a death toll of over 4.2 million in 220 countries ( European Centre for Disease Prevention and Control, 2021). The pandemic has had devastating effects on both public health and societal life ( Rahman & Sathi, 2020; Cori *et al.,* 2020; Parikh *et al.,* 2020). In Africa, about 7,043,648 cases have been reported, with Nigeria accounting for over 183,000 cases and over 2000 in deaths ( Worldometer, 2021). In order to curtail the spread of the virus, the government raised public awareness on preventative health practices, enforced closure of volatile public and private facilities, enforced small-sized gathering with adequate spacing in public spaces, enforced stay at home order in flag point areas and inter and intra-border mobility to put national population statistics under check ( Egbi *et al.,* 2020). Aronu *et al.* (2021) in a cross-sectional study on COVID-19 knowledge and preventive practices among college adolescents in Nigeria, reported that the study participants had a low compliance rate with the enforcements. Many of the young adolescents acknowledged that only the aged and patients with comorbidity are at the high-risk end of COVID-19 mortality. In a study that was conducted in Ethiopia by Kebede *et al.* (2020), they identified that adolescents were less likely to practice preventive measures, and in turn, such behaviours would pose more problems in the preventive processes for COVID-19. Kebede *et al.* (2020b) in another study also stated that the transmission rate of the disease has increased rapidly. In spite of the available vaccines against the virus, the emergence of new strains and variants of the virus continues to pose global health challenges ( Mohammed *et al.*, 2022; van Beek, 2023). According to Bates *et al.* (2021) preventive measures must be adopted to curb the spread of COVID-19. However, it is also important to note that only sufficient knowledge can prompt behavioural changes needed to curb the spread of the COVID-19 ( Van Nhu *et al*., 2020; Belete *et al*., 2021; Khattak *et al.*, 2022). This is especially true in respect to the knowledge, attitude and practice towards the prevention of COVID-19 among the youths ( Umeizudike *et al*., 2021). However, the effects of reported knowledge have not been seen in the attitude and safety practices of some of the youths in South-West Nigeria. Several studies have shown satisfactory levels of knowledge but all these studies are among the adult population ( Kebede *et al.*, 2020b; Abdelhafiz *et al*., 2020; Centers for Disease Control and Prevention, 2021). Several studies also reported, that those with medical-related occupations were more likely to have better knowledge that would also spill into an appropriate preventive attitude and practice ( Noreen *et al*., 2020; Shi *et al.,* 2020). Some studies also considered the limited facts about adherence to the practice of preventive measures of Covid-19 ( Alzoubi *et al.,* 2020; Nazli *et al.,* 2020; Rahman & Sathi, 2020; Kebede *et al.*, 2020a, 2020b; Feyisa, 2021). Another study noted that people with underlying health conditions generally felt the need to take greater preventive approach and attitude towards the virus ( Adesegun *et al.,* 2020; Belete *et al.*, 2021).

In the context of the ongoing COVID-19 pandemic, it is evident that this global public health crisis continues to exert a profound and extensive influence on a global scale ( Ataguba, 2020; Napitu & Sipayung, 2023). The available global statistical data underscores the significant ramifications of the virus, both in terms of its widespread incidence and the lamentable loss of human lives. As nations and regions grapple with the manifold challenges presented by this pandemic, it is pertinent to accord due recognition to the pivotal role played by informed knowledge, attitudes, and preventative practices across various demographic strata globally ( Rahman & Sathi, 2020; Mahfouz & AlQahtani, 2023; Sun *et al.*, 2023). While governmental bodies and health agencies have implemented a spectrum of measures aimed at curbing the virus’s dissemination ( Wang *et al.*, 2023; Kim *et al.*, 2023), encompassing public awareness initiatives ( Yanti *et al.*, 2020) and constraints on mobility ( Zhou *et al.*, 2020), the efficacy of these interventions is fundamentally contingent upon the willingness of individuals, especially the youth demographic, to embrace and steadfastly adhere to the prescribed behavioural protocols. Therefore, there is an urgent need to facilitate more studies on COVID-19 health promotion among Nigerian youths due to paucity of data on the compliance rate of COVID-19 preventative measures.

## Methods

### Participation and procedure

A cross-sectional online survey was anonymously administered online for the young adolescents between the ages of 15 and 26 years who are undergraduates of Afe Babalola University. Afe Babalola University is a private university located in the South-Western region of Nigeria. The institution has almost 8,000 undergraduate students at the time this study was conducted. Only the Afe Babalola University undergraduate students who were healthy, mentally stable and fully residential on campus were eligible to participate in the study. Postgraduates students were excluded from the study. Students were contacted in their various colleges and undergraduate residential hostels to complete the questionnaire ( Jaiyesimi *et al.,* 2022b) *via* a shared link. The links were pasted on the commonly used social media platforms (WhatsApp and Telegram). Students were also encouraged to share the links with each other. A convenience sampling technique was adopted for the study ( Jiang *et al.,* 2021). The sample size was calculated using EPI-info v.7. (EpiData Software) (RRID:SCR_008485). The assumption was set at 50% expected frequency, 95% confidence level, 5% acceptance margin of error, 4.0 design effect with 8000 estimated population size. The final adequate sample for the study was calculated to be 1468 students.

The study was carried out from March 23 - April 25, 2021 when the undergraduate students of Afe Bablola Univeristy were back to school. The COVID-19 knowledge, attitude and practice (KAP) questionnaire was adapted from the study of Ferdous *et al.* (2020). Their study focused on the young adolescents and adults while this study focused on the young adolescents. The questionnaire was administered online to avoid physical contact and to reach more respondents. The questionnaire was designed with Google forms (Google Inc, 2022) and the link was shared *via* social media platform (WhatsApp and Telegram). A total of 1,529 respondents completed the questionnaire in proportion of 626 male (40.9%) and 903 females (59.1%).

### Measures

The validated modified COVID-19 KAP questionnaire ( Elbur *et al.,* 2016; Medani *et al.,* 2018; Al-Hanawi *et al.,* 2020; Ferdous *et al.,* 2020) was tested with Cronbach Alpha for reliability (α = .66) excluding the demographic variables. SPSS software version 25 (IBM corp, 2017) (RRID:SCR_016479) was used to calculate the reliability. Age, gender, family social class, type of residence, region of residence, number of family members, and family status were the demographic variables added to the COVID-19 KAP questionnaire for the purpose of the study considering the selected population. Gender (male and female) and region of residence (south and north) had two sub-categories, while family social class (upper, middle and lower), number of family members (less than 5, 5-10, greater than 10), types of residence (large cities, mid-level cities and small cities) and family status (nuclear, extended and joint) had three sub-categories. Only age had four sub-categories (15-17, 18-20, 21-23 and 24-26 years). Under the KAP section, the subsections for knowledge and attitude comprised six questions each, while the practice section comprised seven questions. No item was removed from the original survey but it was modified (in terms of the grammatical composition of the items) for the purpose of the study.

The knowledge of COVID-19 sub-section had six items on a three-point rating scale (YES, NO and I DON’T KNOW). Each item was assigned a score to rate the response of the respondents (YES = 1, NO = 0, I DON’T KNOW = 0). The maximum summation of all scores on the sub-scale equals six. A cut-off score <4 means LESS ACCURATE, while a cut-off score ≥4 means MORE ACCURATE knowledge on the sub-scale of six. The attitude sub-section three-point rating scale included AGREE, UNDECIDED and DISAGREE with scores assigned respectively (1,0,0). The maximum summation of all scores on the sub-scale equals six. A cut-off score <4 means LESS POSITIVE, while a cut-off score ≥4 means MORE POSITIVE on the sub-scale of six. The practice sub-section also has three-point rating scale (YES = 1, NO = 0, SOMETIMES = 0). The maximum summation of all scores on the sub-scale equals seven. A cut-off score <5 means LESS FREQUENT, while a cut-off score ≥5 means MORE FREQUENT practice on the sub-scale of seven. These cut-off scores were determined to according to a previous study on COVID-19 ( Ferdous *et al.*, 2022).

### Statistical analysis

The obtained responses from the Google form were downloaded *via* the google sheet into the Microsoft Excel (Microsoft, USA, 2019) (RRID:SCR_016137) for data cleaning. Incomplete responses with valid missing values were excluded from the datasheet. The descriptive statistics of frequency counts and percentages were computed for the variables used in the study while chi-square cross-tabulation were computed to test the significant association between the demographic variables and the COVID-19 KAP. SPSS (RRID:SCR_002865) was used to calculate the reliability and chi-square cross-tabulation for the study.

## Results

A total of 1,529 respondents with the age ranges from 15–26 years voluntarily participated in the study ( Jaiyesimi *et al.,* 2022a). Most of the respondents (76.6%) fall within the category of 15–17 years. More females were involved in the study (59.1%) than male (40.9%). Most of the respondents were from nuclear (87%) and perceived middle-class family social status (79.2%), with a range of 5–10 family members (76.3%), lived in metropolitan cities (52.2%) within southern region of Nigeria (70.7%) ( Table 1).

**Table 1.** Demographic characteristics of participants (N = 1529).

Variables	Frequency(%)
Gender	
Female	903(59.1)
Male	626(40.9)
Age	
15-17	1181(77.2)
18-20	323(21.1)
21-23	20(1.3)
24-26	5(0.3)
Perceived family social status	
Upper	248(16.2)
Middle	1211(79.2)
Lower	70(4.6)
Region of residence	
South	1081(70.7)
North	448(29.3)
Number of family members	
Less than 5	260(17)
5-10	1167(76.3)
Greater than 10	102(6.7)
Type of residence	
Large cities	798(52.2)
Mid-level cities	629(41.1)
Small cities	102(6.7)
Family status	
Nuclear	1330(87)
Extended	77(11.6)
Joint	22(1.4)

N, Total number of samples; %, Percentages.

### Perception towards COVID-19 mode of transmission, symptoms, risk factors, treatments, preventive measures, and challenges

As shown in Table 2, more than half of the respondents (54.2%) identified that COVID-19 could easily spread through close contact with an infected person, while less than 2% claimed ignorance of the mode of transmission of the viral infection. Most of the respondents (90.1%) acknowledged that the incubation period of the virus is within 2-14 days. Almost all the respondents (98.8%) identified fever, dry cough and difficult breathing as the symptoms of the viral infection. More than half of the respondents reported that the population group at most risk of the virus is the elderly (57.9%) followed by the individuals with chronic conditions such as cancer, diabetes, respiratory infections and so on. Pregnant women (0.6%), children (2%), migrants (5.2%) were not considered as major risk groups by most of the participants. Most of the respondents reported that vaccination (62.8%) and supportive treatment (33.7%) are methods of treating COVID-19. Most of the respondents identified social distancing (34.3%), washing of hands (21.8%) and use of nose or face mask (15.5%) as the most effective COVID-19 preventive measures. More than half of the respondents recognized home-based handwashing with soap (59.1%) and restrictions of visitors (37.5%) as effective measures to protect the family against the spread. Almost 50% of the respondents reported no problem in creating awareness against the spread, however, 20.7% could not limit outdoor life and preferred not to use a protective face mask.

**Table 2.** Perception towards COVID-19 mode of transmission, symptoms, risk factors, treatments, preventive measures, and challenges.

Variables	Total (Number=1529)
In what ways does COVID-19 spread?	
Coughing directly transmits the virus	444(29)
Contact with contaminated surfaces	233(15.2)
Having close contact with someone who is infected	828(54.2)
Don’t know	24(1.6)
**What are the intervals of COVID-19 symptoms?**	
2–5 days	102(6.7)
2–14 days	1378(90.1)
Don’t know	49(3.2)
**Tick the COVID-19 symptoms from the list below?**	
Fever, cough, shortness of breath	1511(98.8)
Sore throat, blocked nose	8(0.5)
Headache	2(0.1)
Diarrheal	2(0.1)
Don’t know	6(0.4)
**Tick those who are at most risk of COVID-19?**	
Aged persons	886(57.9)
Pregnant mothers	10(0.7)
Children	30(2)
Individuals with chronic diseases	497(32.5)
People from COVID-19 infected regions	79(5.2)
Don’t know	27(1.8)
**Tick the most effect COVID-19 treatment that you know**	
Medical care	516(33.7)
Vaccine	960(62.8)
Don’t know	53(3.5)
**Tick the most effective method of preventing COVID-19**	
Handwashing	333(21.8)
No dirty contact with eyes and nose	101(6.6)
No contact with persons infected with COVID-19	139(9.1)
Wearing nose mask	237(15.5)
Keeping 1m or more of social distance	524(34.3)
Self-quarantine	71(4.6)
Taking all family members in home quarantine	13(0.9)
Adequate provision of healthcare facilities and supplies	32(2.1)
Raising awareness against COVID-19	79(5.2)
**Tick measures taking to safeguard the family members against COVID-19**	
Restriction on the number of guests at home per time	574(37.5)
Handwashing equipment at the entrance of the house	903(59.1)
Handwashing after touching pets	52(3.4)
**Tick what represent the attitude of your family towards COVID-19**	
Negligence towards the COVID-19 preventive practices	137(9)
Unwilling to wear the nose or face mask	316(20.7)
Not being able to stop going out of the house	316(20.7)
Don't face the problem	760(49.7)

%, Percentages, COVID-19, coronavirus disease 2019.

### Knowledge of COVID-19

As shown in Table 3, no items under the knowledge of COVID-19 had significant association based on gender distribution. Only participants with a family of 5–10 members showed significant association with knowledge accuracy on COVID-19 (χ ^2^ = 6.077, p = 0.044). Furthermore, those in this group had 80.8% more accurate knowledge of COVID-19 than respondents from other family categories (see Table 5). The respondents from metropolitan cities reported the highest percentage of accurate knowledge (53.3%), followed by mid-level cities (41.6%) and the small cities’ respondents had the least response (5.1%). Moreover, those who are from the middle family social class reported more accurate knowledge (76.3%) than others in the upper (18.9%) and lower class (4.8%) (see Table 6).

**Table 3.** Knowledge of COVID-19 based on gender differences.

Variables	Total (N=1529)	Female	Male	χ ^2^	df	p-value
	Frequency(%)	Frequency(%)	Frequency(%)			
Do you know that COVID-19 is a life-threatening disease?						
Yes	1492(97.6)	881(59)	611(41)	.115	2	0.940
No	26(1.7)	15(57.7)	11(42.3)			
Don't know	11(0.7)	7(63.6)	4(36.4)			
Do you know that COVID-19 can only affect human beings?						
Yes	653(42.7)	367(56.2)	286(43.8)	3.986	2	0.140
No	714(46.7)	439(61.5)	275(38.5)			
Don't know	162(10.6)	97(59.9)	65(40.1)			
Do you know that an animal can be infected by a human being?						
Yes	534(34.9)	306(57.3)	228(42.7)	1.147	2	0.560
No	621(40.6)	375(60.4)	246(39.6)			
Don't know	374(24.5)	222(59.4)	152(40.6)			
Do you know that a human being can be infected by animal?						
Yes	790(51.7)	460(58.2)	330(41.8)	.883	2	0.640
No	440(28.8)	268(60.9)	172(39.1)			
Don't know	299(19.6)	175(58.5)	124(41.5)			
Can COVID-19 spread through contact or consumption of animal products?						
Yes	481(31.5)	273(56.8)	208(43.2)	3.816	2	0.150
No	688(45)	425(61.8)	263(38.2)			
Don't know	360(23.5)	205(56.9)	155(43.1)			
Can COVID-19 spread through contact or consumption of well-cooked food?						
Yes	88(5.8)	53(60.2)	35(39.8)	3.720	2	0.160
No	1235(80.8)	741(60)	494(40)			
Don't know	206(13.5)	109(52.9)	97(47.1)			

χ ^2^, Chi-square; df, degree of freedom; p-value, significant level; %, percentage, COVID-19, coronavirus disease 2019; N, Total number of samples.

### Attitude towards COVID-19

According to Table 4, there was a significant association between gender and the need to report the suspected case of COVID-19 infection to the health authorities (χ ^2^ = 14.075, p = 0.001). Females (58.7%) are more likely to report a suspected case of COVID-19 than their male counterparts (41.3%).

**Table 4.** Attitude towards COVID-19 based on gender differences.

Variables	Total (N=1529)	Female	Male	χ ^2^	df	p-value
	Frequency(%)	Frequency(%)	Frequency(%)			
A suspected COVID-19 case should be reported immediately						
Agree	1462(95.6)	858(58.7)	604(41.3)	14.075	2	0.001 *
Undecided	25(1.6)	10(40)	15(60)			
Disagree	42(2.7)	35(83.3)	7(16.7)			
Face or nose mask should be used in public spaces						
Agree	1516(99.1)	895(59)	621(41)	3.699	2	0.157
Undecided	6(0.4)	2(33.3)	4(66.7)			
Disagree	7(0.5)	6(85.7)	1(14.3)			
Handwashing should be practice at all times especially in public spaces						
Agree	1505(98.4)	890(59.1)	615(40.9)	2.903	2	0.234
Undecided	11(0.7)	4(36.4)	7(63.6)			
Disagree	13(0.9)	9(69.2)	4(30.8)			
COVID-19 is a preventable disease						
Agree	1423(93.1)	835(58.7)	588(41.3)	4.279	2	0.118
Undecided	85(5.6)	51(60)	34(40)			
Disagree	21(1.4)	17(81)	4(19)			
It can be treated at home						
Agree	440(28.8)	261(59.3)	179(40.7)	.095	2	0.954
Undecided	319(20.9)	186(58.3)	133(41.7)			
Disagree	770(50.4)	456(59.2)	314(40.8)			
Health education can play an important role in COVID-19 prevention						
Agree	1517(99.2)	898(59.2)	619(40.8)	1.582	2	0.453
Undecided	10(0.7)	4(40)	6(60)			
Disagree	2(0.1)	1(50)	1(50)			

χ ^2^, Chi-square; df, degree of freedom; p-value, significant level; %, percentage, COVID-19, coronavirus disease 2019; N, Total number of samples.

* Significant p-value.

Other items on the scale were not significantly associated with gender, even though females (58.3%) showed more positive attitude than male (41.7%) (see Table 6). The respondents from the metropolitan cities had more significant positive attitude (χ ^2^ = 12.191, p = 0.002, 57.1%), than other city-dwellers (mid-level = 36.9% and small cities = 6%) (see Table 6).

### Practice of COVID-19 preventive measures

Items relating to the practice of COVID-19 preventive measures based on gender distribution are presented in Table 5. The question related to the use of handkerchief during coughing/sneezing had a significant association based on gender distribution (χ ^2^ = 7.993, p = 0.018) with higher compliance from female participants (61.2%). Frequent washing of hands to curb the spread was significantly (χ ^2^ = 16.895, p = 0.000) more common in female (61.4%) than male respondents (38.6%). Significantly more females (59.2%) were (χ ^2^ = 8.904, p = 0.012) involved in the practice of social distancing or home quarantine as a preventive measure than male respondents (40.8%). In following all the government rules concerning the prevention of COVID-19, majority of the females (59.4%) were significantly (χ ^2^ = 10.260, p = 0.006) more compliant with the rules (see Table 5) than male respondents (40.6%). The cross-tabulation for demographic variables (see Table 6) showed that the participants from middle class family were significantly more involved in the preventive measures (76.4%, χ ^2^ = 8.115, p = 0.017) than other family classes (upper (19.2%) and lower (4.5%)).

**Table 5.** Practice against the spread of COVID-19 based on gender differences.

Variables	Total (N=1529)	Female	Male	χ ^2^	df	p-value
	n(%)	n(%)	n(%)			
Do you use tissues or handkerchief during coughing/sneezing?						
Yes	948(62)	580(61.2)	368(38.8)	7.993	2	0.018 *
No	122(8)	59(48.4)	63(51.6)			
Sometimes	459(30)	264(57.5)	195(42.5)			
Do you wash hands frequently using water and soaps?						
Yes	1137(74.4)	698(61.4)	439(38.6)	16.895	2	0.000 *
No	64(4.2)	24(37.5)	40(62.5)			
Sometimes	328(21.5)	181(55.2)	147(44.8)			
Do you avoid touching face and eyes?						
Yes	896(58.6)	543(60.6)	353(39.4)	4.043	2	0.132
No	168(11)	88(52.4)	80(47.6)			
Sometimes	465(30.4)	272(58.5)	193(41.5)			
Do you maintain social distance (or home quarantine)?						
Yes	1074(70.2)	636(59.2)	438(40.8)	8.904	2	0.012 *
No	106(6.9)	49(46.2)	57(53.8)			
Sometimes	349(22.8)	218(62.5)	131(37.5)			
Do you eat healthy food focusing on outbreak?						
Yes	1129(73.8)	648(57.4)	481(42.6)	4.949	2	0.084
No	139(9.1)	88(63.3)	51(36.7)			
Sometimes	261(17.1)	167(64)	94(36)			
Do you maintain a healthy lifestyle focusing on outbreak?						
Yes	1155(75.5)	677(58.6)	478(41.4)	.394	2	0.821
No	100(6.5)	60(60)	40(40)			
Sometimes	274(17.9)	166(60.6)	108(39.4)			
Do you obey all government rules related to the COVID?						
Yes	1075(70.3)	639(59.4)	436(40.6)	10.260	2	0.006 *
No	86(5.6)	37(43)	49(57)			
Sometimes	368(24.1)	227(61.7)	141(38.3)			

χ ^2^, Chi-square; df, degree of freedom; p-value, significant level; %, percentage, COVID-19, coronavirus disease 2019; N, Total number of samples.

* Significant p-value.

**Table 6.** Association between demographic variables and levels of KAP towards COVID.

Variables	Knowledge		χ ^2^	P-value	Attitude		χ ^2^	P-value	Practice		χ ^2^	P-value
	Less accurate	More accurate			Less positive	More positive			Less frequent	More frequent		
	Frequency (%)	Frequency (%)			Frequency (%)	Frequency (%)			Frequency (%)	Frequency (%)		
Gender												
Female	710(59.4)	193(57.8)	.287	0.592	502(59.7)	401(58.3)	.309	0.578	483(57.8)	420(60.5)	1.121	0.290
Male	485(40.6)	141(42.2)			339(40.3)	287(41.7)			352(42.2)	274(39.5)		
Type of residence												
Metropolitan cities	620(51.9)	178(53.3)	1.725	0.422	405(48.2)	393(57.1)	12.191	0.002 *	416(49.8)	382(55)	4.242	0.120
Mid-level cities	490(41)	139(41.6)			375(44.6)	254(36.9)			362(43.4)	267(38.5)		
Small cities	85(7.1)	17(5.1)			61(7.3)	41(6)			57(6.8)	45(6.5)		
Number of family members class												
Less than 5	218(18.2)	42(12.6)	6.077	0.044 *	131(15.6)	129(18.8)	3.748	0.154	157(18.8)	103(14.8)	4.483	0.106
5-10	897(75.1)	270(80.8)			648(77.1)	519(75.4)			621(74.4)	546(78.7)		
Greater than 10	80(6.7)	22(6.6)			62(7.4)	40(5.8)			57(6.8)	45(6.5)		
Family social class												
Upper	185(15.5)	63(18.9)	2.322	0.313	144(17.1)	104(15.1)	1.121	0.571	115(13.8)	133(19.2)	8.115	0.017 *
Middle	956(80)	255(76.3)			659(78.4)	552(80.2)			681(81.6)	530(76.4)		
Lower	54(4.5)	16(4.8)			38(4.5)	32(4.7)			39(4.7)	31(4.5)		

χ ^2^, Chi-square; df, degree of freedom; p-value, significant level; %, percentage, COVID-19, coronavirus disease 2019; N, Total number of samples; KAP, knowledge, attitude and practice.

* Significant p-value.

## Discussion

This research was carried out among Nigerian educated youth in order to find out their perception towards COVID-19 mode of transmission, symptoms, risk factors, treatments, preventive measures, and challenges. The study examined the knowledge, attitude, and practice towards COVID-19 and their association with the selected sociodemographic variables of the respondents.

In the perception domain, the participants showed good knowledge about the mode of transmission of the virus as well as the incubation period. This finding was similar to a study carried out by Peng *et al.* (2020) among undergraduates in China, who also demonstrated good knowledge of mode of transmission and incubation period of COVID-19 infection. However, this finding was in contrast with a similar study carried out in Congo, where respondents demonstrated poor knowledge of COVID-19 mode of transmission ( Carsi Kuhangana *et al.,* 2020). The difference in socio-economic status and educational level were identified as key factors as the latter was conducted among sellers and customers who patronised some big public markets in Congo, unlike the current study where the respondents were entirely young students. The majority of the respondents were familiar with the symptoms of COVID-19 and also reported the elderly as the most vulnerable group, followed by individuals with co-morbid medical conditions. This finding was similar to a study that was conducted in Jimma town, Ethiopia, where larger percentage of the participant were aware of the key clinical symptoms and the groups at high risk of developing a severe form of the disease ( Kebede *et al.,* 2020b). The similarity in findings could be as a result of the efforts of the health and educational agency of the government and access to information from social (WhatsApp and Telegram) and broadcast media (television and radio) by the study participants as reported in the current study.

Most of the respondents identified that vaccination, followed by supportive treatment have important roles to play in the management of COVID-19 disease. This was in marked contrast to the findings of ( Ferdous *et al.,* 2020) where only 1% of the participant reported vaccination as having a role in the prevention of COVID-19 infection. This disparity could be due to the fact that vaccine development was still at a rudimentary stage around the end of first quarter of 2020 when the study was conducted. However, in a similar study conducted around the same time, Srichan *et al.* (2020) found that 31.2% were aware of the vaccine as a potential option. Most of the respondents recognized home-based handwashing with soap and water, followed by restriction of visitors as measures to protect the family against COVID-19 virus. They also identified social distancing and use of face masks as preventive measures. These findings agree with that of many other studies which reported good perception of hand hygiene, social distancing, wearing face mask and avoiding crowded areas ( Akalu *et al.,* 2020; Asmelash *et al.,* 2020; Kassie *et al.,* 2020; Tamire *et al.,* 2020). However, in an earlier study, Ferdous, *et al.* (2020) reported that most of the respondents indicated negligence about the severity of the disease, reluctance to use face mask and not being able to control going out of the house. Understandably, this study was conducted immediately after lockdown was instituted in the study area and the populace could still be struggling with the physical, social and emotional barriers and limitations posed by the lockdown.

The respondents reportedly have sufficient knowledge of the virus because of exposure to the media and internet, coupled with some health-related courses in the university that focused mainly on trending public health issues. A similar result was carried out by Salman *et al.* (2020) among Pakistani University students in May 2020. The study found out that the students had sufficient knowledge and a positive attitude towards COVID-19. They are knowledgeable of the causative agent, incubation period, the spread, and preventive measures ( Salman *et al.,* 2020). The depth of the knowledge can also be associated with the fact that the respondents in their study and the current study were students. According to the current study, knowledge of COVID-19 could be a possible determinants of respondents’ attitude and practice towards the disease. This is unlike the study that was carried out by Bhagavathula *et al.* (2020), where it was discovered that the students had insufficient knowledge about the virus although they had a positive attitude towards the prevention of the spread of the virus.

A positive attitude was also exhibited by the majority of our respondents, they indicated that they were willing to adhere to the various measures put in place by various units or groups to curb the spread of the infection. A notable measure is their responsibility towards the University Hospital isolation centre, students have continued to be very observant and helpful in assisting the University to report any suspected cases to the isolation centre for clinical testing. A similar positive attitude was observed in researches carried out in Malaysia and China ( Azlan *et al.,* 2020; Zhong *et al.,* 2020). Positive attitude towards hand hygiene, social distancing, use of face masks and avoiding crowded area prevented the spread of the virus ( Umeizudike *et al.,* 2021). Most of the respondents (70.3%) in this study reported that they adhere to the Government rules on the prevention of COVID-19 (see Table 5). In the current study, participants from middle-class families were more engaged in preventive practices than other categories of family social status (upper and lower). Due to the homogeneity of the selected sample (students of the same university) and the selected age category, the study cannot be generalised for the entire population.

## Conclusions

This study showed evidence of high prevalence of knowledge related to COVID-19 in majority of the study participants. A significant proportion of the respondents understood the basic information, possessed positive attitude and demonstrated proactive practices towards COVID-19 infection. This is a pointer to the efficacy and success of present public health campaigns. However, male respondents showed poorer COVID-19 preventive practices compared to their female counterpart, and respondents from upper and lower socio-economic class demonstrated less-than-adequate knowledge, attitude and practices towards COVID-19. Public health interventions should therefore be tailored along these lines.

### Limitations of the study

The scope of the study was limited by the data collection procedure. The participants were enrolled *via* Whatsapp and Telegram, due to the need capture the calculated sample size, which limited the study to convenience sampling technique. Some potential respondents were, probably, not captured due to irregular internet access. Those who did not have access to the selected social media platform might be unknowingly exempted from the study. The choice of population (undergraduate students from a university) might have exposed the study to selection bias which limits the generalizability of the study. Considering that the selected university is private institution, further study should focus on the undergraduate students at a public institution.

## Data availability

### Underlying data

Figshare: COVID-19 Knowledge and symptoms July 13th.xlsx, https://doi.org/10.6084/m9.figshare.20341098 ( Jaiyesimi *et al.,* 2022a).

This project contains the following underlying data:

- COVID-19 Knowledge and symptoms July 13th.xlsx (Raw de-identified questionnaire answers).

### Extended data

Figshare: The Nigerian youth and their positive attitude towards the national drive against the spread of COVID-19: A cross-sectional online survey, https://doi.org/10.6084/m9.figshare.20379930.v1 ( Jaiyesimi *et al.,* 2022b).

This project contains the following extended data:

- COVID-19 Knowledge and symptoms July 10th Questionnaire.docx

Data are available under the terms of the Creative Commons Attribution 4.0 International license (CC-BY 4.0).

## Ethical approval

Ethical approval was obtained from the Health Research Ethics Committee (ABUAD/HREC/04/10/829). The participants under the age of 18 (duly registered as undergraduate student of the university) are covered by the institutional policy to voluntarily participate in this study with due consideration of no physical harm to the participants.

## Consent to participate

All respondents gave written informed consent to participate in the research through informed written consent.
