# Supplementary material for: The Nigerian youth knowledge, Attitude and Practice (KAP) towards the national drive against the Spread of COVID-19: An online cross-sectional Survey
Source: F1000Res. 2024 Jan 9;11:1353. Originally published 2022 Nov 21. [Version 2] doi: 10.12688/f1000research.121826.2 (PMC10988216; doi:10.12688/f1000research.121826.2)
Supplement: Peer review history

# Reviewer report: Reviewer response for version 2

**DOI:** 10.5256/f1000research.157254.r252109

**Published:** 2024-04-02

**Authors:** Mohammad Daud Ali

**Affiliations:** 1 Department of Pharmacy, Mohammed Al-Mana College for Medical Sciences, Dammam, Saudi Arabia

Comments:

1. The introduction is poorly described and not aligned with the study objective
2. Kindly add the rationale of the study under the last paragraph of the introduction
3. Methodology is poorly described kindly rewrite
4. Kindly write the study site
5. Kindly add inclusion and exclusion criteria for the study
6. How sample size determined for the study
7. Kindly how data were collected for the study
8. Kindly add study duration
9. How selection biases were minimized
10. Discussion needs to update and compare findings with the latest published article
11. Kindly add limitations of the study
12. Please scan your manuscript again and correct some topological errors
13. Please make sure your manuscript was written in line with this journal submission guideline

Is the work clearly and accurately presented and does it cite the current literature?

Partly

If applicable, is the statistical analysis and its interpretation appropriate?

I cannot comment. A qualified statistician is required.

Are all the source data underlying the results available to ensure full reproducibility?

Partly

Is the study design appropriate and is the work technically sound?

Partly

Are the conclusions drawn adequately supported by the results?

Partly

Are sufficient details of methods and analysis provided to allow replication by others?

Partly

Reviewer Expertise:

Dr. Mohammad Daud Ali. Pharmacy Practice & Clinical Pharmacy.

I confirm that I have read this submission and believe that I have an appropriate level of expertise to confirm that it is of an acceptable scientific standard, however I have significant reservations, as outlined above.

# Reviewer report: Reviewer response for version 2

**DOI:** 10.5256/f1000research.157254.r236425

**Published:** 2024-02-27

**Authors:** Pathiyil Ravi Shankar

**Affiliations:** 1 IMU Centre for Education, International Medical University, Kuala Lumpur, Malaysia

The authors have addressed my comments.

Is the work clearly and accurately presented and does it cite the current literature?

Partly

If applicable, is the statistical analysis and its interpretation appropriate?

I cannot comment. A qualified statistician is required.

Are all the source data underlying the results available to ensure full reproducibility?

Yes

Is the study design appropriate and is the work technically sound?

Yes

Are the conclusions drawn adequately supported by the results?

Yes

Are sufficient details of methods and analysis provided to allow replication by others?

Yes

Reviewer Expertise:

Public health. COVID-19. KAP studies

I confirm that I have read this submission and believe that I have an appropriate level of expertise to confirm that it is of an acceptable scientific standard.

# Reviewer report: Reviewer response for version 1

**DOI:** 10.5256/f1000research.133729.r174663

**Published:** 2023-07-11

**Authors:** Saadullah Khattak

**Affiliations:** 1 Henan International Joint Laboratory for Nuclear Protein Regulation, Henan University, Kaifeng, Henan, China

Thank you for sharing this interesting article The Nigerian Youth and their positive attitude towards the national drive against the Spread of COVID-19: A cross-sectional online Survey. Here are some suggested edits and comments that could help to improve the article:

**Major revision**

- Write a title such as The Nigerian youth knowledge, Attitude and Practice (KAP) Towards the national drive against the Spread of COVID-19: A cross-sectional online Survey or The Nigerian youth knowledge, Attitude and Practice (KAP) Towards the COVID-19: A cross-sectional online survey.
- Revise and summarize the abstract in 250 words.
- The full manuscript needs to revise to remove grammatical and minor. An error such as first mentioning the complete form and then abbreviation for “COVID-19, and KAP”, etc.,
- Full stop after the citation and someplace before the citation. Please use the same pattern.
- Most of the cited references are from 2019-2021. Please also cite some recent work from 2021 onwards.
- Section: Introduction and discussion content can be enriched with research articles and more. For example:

DOI: 10.3390/vaccines10101583, DOI: 10.3390/ijerph19137926, DOI: 10.29052/IJEHSR.v10.i2.2022.255-272, DOI: 10.3389/fmed.2021.747819, DOI: 10.3390/biom11060912

- *Change the young adolescent word with the university student, youth, etc*
- *Please explains the reason to start with 15 years age.*
- Please explains the reason (Postgraduates students were excluded from the study)—exclusion criteria.
- The survey contained any informed consent pages?
- The language of the questionnaire?
- The survey was done through Telegram and WhatsApp. One bias is it is limited to people who have these social media accounts, and authors mention this bias. However, how mobile numbers were selected for the WhatsApp channel is unclear. This needs to be clarified.
- Finding sections needs improvement, and authors need to explain statistical analysis results rather than the same info available in the tables.
- Authors should use other statistical tools or weighted averages.
- The rationale of the study is not clear. I am not sure why the authors have conducted the study when similar investigations are available from Nigeria. The authors need to clarify the rational of this study in the introduction section.
- The questionnaire was developed from previous studies, but there is no information on the data collection form's validation, reliability, and translation.
- I appreciate the efforts of the authors to have a large sample size for this study, but this study needs extensive revisions and even re-analysis.

Is the work clearly and accurately presented and does it cite the current literature?

Partly

If applicable, is the statistical analysis and its interpretation appropriate?

Yes

Are all the source data underlying the results available to ensure full reproducibility?

Yes

Is the study design appropriate and is the work technically sound?

Partly

Are the conclusions drawn adequately supported by the results?

Yes

Are sufficient details of methods and analysis provided to allow replication by others?

Yes

Reviewer Expertise:

NA

I confirm that I have read this submission and believe that I have an appropriate level of expertise to confirm that it is of an acceptable scientific standard, however I have significant reservations, as outlined above.

## References

[1] : Assessment of Attitudes and Intentions towards COVID-19 Vaccines and Associated Factors among General Populations of Pakistan: A Cross-Sectional Study. *Vaccines (Basel)* .2022;10(10) : 10.3390/vaccines10101583 10.3390/vaccines10101583 36298447 PMC9609017

[2] : Knowledge, Attitude, and Perception of Cancer Patients towards COVID-19 in Pakistan: A Cross-Sectional Study. *Int J Environ Res Public Health* .2022;19(13) : 10.3390/ijerph19137926 10.3390/ijerph19137926 35805584 PMC9265320

[3] : Drugs being tested against covid-19 to slow down its spread and find effective treatment: A systematic review. *INTERNATIONAL JOURNAL OF ENDORSING HEALTH SCIENCE RESEARCH (IJEHSR)* .2022;10(2) : 10.29052/IJEHSR.v10.i2.2022.255-272 255-272 10.29052/IJEHSR.v10.i2.2022.255-272

[4] : Assessment of General Populations Knowledge, Attitude, and Perceptions Toward the Coronavirus Disease (COVID-19): A Cross-Sectional Study From Pakistan. *Front Med (Lausanne)* .2021;8: 10.3389/fmed.2021.747819 747819 10.3389/fmed.2021.747819 35036408 PMC8754052

[5] : Genome-Wide Analysis of Codon Usage Patterns of SARS-CoV-2 Virus Reveals Global Heterogeneity of COVID-19. *Biomolecules* .2021;11(6) : 10.3390/biom11060912 10.3390/biom11060912 34207362 PMC8233742

# Reviewer report: Reviewer response for version 1

**DOI:** 10.5256/f1000research.133729.r171237

**Published:** 2023-05-09

**Authors:** Pathiyil Ravi Shankar

**Affiliations:** 1 IMU Centre for Education, International Medical University, Kuala Lumpur, Malaysia

Abstract section: Methods subsection, what do you mean by a young adolescent?

 Introduction: Toward the end of the Introduction, kindly mention why this study among adolescents is necessary.

 Methods:

- Can students enter university at the age of 15 years? Can you explain university admissions in Nigeria? In most countries, students enter university only after the age of 17.
- Table 1: What is meant by place of residence – South, North as all respondents were staying in the university hostels? I believe you are referring to where their family is staying.
- Middle, upper, and lower may be better characterized as economic status rather than social status, though these may be linked.
- Page 4, paragraph 1: What do you mean by the grammatical composition of the items?
- In the results section, only differences in scores according to gender are shown. What about other demographic characteristics?
- Do university fees and living costs limit students from lower socioeconomic classes from attending the university?
- It is nice that the data about the study is publicly available.
- Is the extended data already published? Is there any overlap with the data presented in this manuscript?

Is the work clearly and accurately presented and does it cite the current literature?

Partly

If applicable, is the statistical analysis and its interpretation appropriate?

I cannot comment. A qualified statistician is required.

Are all the source data underlying the results available to ensure full reproducibility?

Yes

Is the study design appropriate and is the work technically sound?

Yes

Are the conclusions drawn adequately supported by the results?

Yes

Are sufficient details of methods and analysis provided to allow replication by others?

Yes

Reviewer Expertise:

Public health. COVID-19. KAP studies

I confirm that I have read this submission and believe that I have an appropriate level of expertise to confirm that it is of an acceptable scientific standard, however I have significant reservations, as outlined above.
